# Silencing TUBB3 Expression Destroys the Tegument and Flame Cells of *Echinococcus multilocularis* Protoscoleces

**DOI:** 10.3390/ani12182471

**Published:** 2022-09-19

**Authors:** Qiqi Shi, Congshan Liu, Lele Huo, Yi Tao, Haobing Zhang

**Affiliations:** National Institute of Parasitic Diseases, Chinese Center for Disease Control and Prevention (Chinese Center for Tropical Diseases Research), NHC Key Laboratory of Parasite and Vector Biology, WHO Collaborating Centre for Tropical Diseases, National Center for International Research on Tropical Diseases, Shanghai 200025, China

**Keywords:** *Echinococcus multilocularis*, RNAi, microtubule, TUBB3, tegument, flame cell

## Abstract

**Simple Summary:**

*Echinococcus multilocularis* is a fox and carnivore tapeworm with important zoonotic potential, as the larval stage can cause alveolar echinococcosis in both humans and livestock. An understanding of the molecular mechanisms of parasite growth and development is essential for disease diagnosis, management and control. This study investigates the potential role of *E. multilocularis* TUBB3 in protoscoleces through RNA interference in vitro with specific short interfering RNAs. Our results show that EmTUBB3 might contribute to tegument formation and protonephridial system flame cell integrity in *E. multilocularis* protoscoleces.

**Abstract:**

Alveolar echinococcosis (AE), caused by infection with the larvae of *Echinococcus multilocularis*, is a neglected tropical disease and zoonosis that causes remarkable morbidity in humans and has economic importance in the livestock industry worldwide. The growth of this parasite resembles the invasion and proliferation of malignant tumours. Microtubules, especially the β-tubulin subunit in the exposed end, are the targets of many antitumour drugs. However, the role of TUBB3, which is the most studied isotype in solid tumours and is also a marker of biological aggressiveness associated with the modulation of tumour metastatic abilities in the growth and development of platyhelminths, is unknown. In this study, protoscoleces (PSCs) are cultivated in monophasic medium in vitro. Using electroporated short interfering RNA (siRNA), EmTUBB3 knockdown was performed with two EmTUBB3-specific siRNAs (siRNA-1 and siRNA-2). qRT–PCR was performed to detect the expression of TUBB3. PSCs viability and the evagination rate and number of body contractions were quantified under a light microscope. Scanning electron microscopy (SEM) and transmission electron microscopy (TEM) were used to observe the ultra-morphological changes of the parasites. After siRNA interference, the EmTUBB3 expression in *E. multilocularis* PSCs was significantly reduced. Reduced viability, a decreased evagination rate and a decreased number of body contractions were also documented. In particular, shrinkage and roughness of the tegument were observed. Ultrastructural changes included marked damage to flame cells, cracked cilia structures enclosed in the cell body and ruptured microtubule structures. EmTUBB3 possibly plays a crucial role in tegument and flame cell integrity in *E. multilocularis* PSCs. Novel drugs targeting this specific beta-tubulin isotype in *E. multilocularis* are potential methods for disease control and deserve further attention.

## 1. Introduction

Infection with the larval stage of *Echinococcus multilocularis,* a fox tapeworm, causes alveolar echinococcosis (AE) in both humans and livestock. As one of the most lethal helminthiases, AE represents an economic and public health problem in many developing countries [1], with an estimated global burden of 666,434 disability-adjusted life years (DALYs) [2]. In epidemic areas, the annual AE incidence ranges from 0.03 to 1.2 per 100,000 population [3]. In addition, the mortality rate in untreated or inadequately treated AE patients within 10 to 15 years of diagnosis is higher than 90% [4]. Hence, echinococcosis has been listed as a neglected disease targeted for control or elimination by 2050 (http://whqlibdoc.who.int/hq/2012/WHO_HTM_NTD_2012.1_eng.pdf accessed on 1 January 2021).

To date, echinococcosis treatment is primarily characterized by the surgical removal of metacestodes and the administration of chemotherapy using benzimidazoles (BZs) [5]. Due to the fact that the early stages of echinococcus infections are typically asymptomatic, most patients seeking medical care are in the late stages of AE; at this stage, patients can be treated with only long-term chemotherapy [6]. However, BZs have limited efficacy against the causative agents of echinococcosis, and some patients show an intolerance to BZs (severe adverse effects); drug resistance to BZs among helminths is common as well [6]. Therefore, given the limitations of the current diagnostic procedures, the toxicity and poor efficacy of available drugs and the challenges in control and prevention, improved diagnostic techniques and novel drug development are urgently needed.

The main mechanism of action of BZs against helminths involves direct binding to β-tubulin, a tubulin heterodimer, which inhibits the polymerization of microtubules, essential elements of all eukaryotic cells [7,8]. BZs exhibit a higher affinity for β-tubulins in helminths than for those in mammals, which could be attributed to differences in their primary amino acid sequences [7,8]. Brehm et al. reported three β-tubulin genes from *E. multilocularis* and revealed that TUBB2 displays several characteristics of a β-tubulin with diminished BZ affinity, while TUBB1 and TUBB3 are structurally related to BZ-sensitive isotypes [7]. In addition, TUBB3 is mostly associated with malignant tumour features, such as tumour growth [9,10], migration and invasion [10,11] and poor outcomes [11,12,13]. Moreover, the differential expression of tubulin isotypes has been extensively associated with the response to microtubule-targeting drugs such as taxanes, commonly used in chemotherapy (reviewed in [14]). The origin of this link may be the known regulation of microtubule dynamics by specific tubulin isotypes, with microtubules containing TUBB3 being more dynamic than other β-tubulin isotypes [15,16,17]. However, the biological characteristics and functions of TUBB3 in helminth parasites remain unknown. Brehm et al. showed that TUBB3 displayed a higher expression in metacestode (containing protoscoleces) than other developmental stages (oncosphere and adult worms) [18]. Hence, we characterized TUBB3 in the protoscoleces (PSCs) of *E. multilocularis*, a type of metacestode, using RNA interference (RNAi).

RNAi is a classic method for the exploration of gene function in parasitic helminths, including flatworms [19,20,21] and nematodes [22,23,24]. However, few studies have been conducted in cestodes [25,26,27,28,29]. In the present study, we used RNAi carried out by electroporation [30] to investigate the role of TUBB3 in *E. multilocularis* PSCs in vitro, and the viability and morphological and biological changes were evaluated.

## 2. Materials and Methods

### 2.1. Parasite Preparation and Culture

Metacestodes of *E. multilocularis* were continuously maintained in Mongolian gerbils (*Meriones unguiculatus*) via intraperitoneal infection with homogenized tissue. At 6–8 weeks post-infection, parasite tissue was isolated and homogenized in phosphate-buffered saline (PBS) containing 100 U/mL penicillin and 100 μg/mL streptomycin. The suspension was filtered through 150-μm gauze to remove debris, followed by filtration through 30-μm gauze to collect PSCs. The PSCs were washed three times with PBS (pH 7.5) and transferred to a RPMI 1640 medium supplemented with 10% foetal bovine serum (FBS), 100 U/mL penicillin and 100 μg/mL streptomycin (HyClone) maintained at 37 °C in an atmosphere of 5% CO_2_.

### 2.2. siRNA Preparation

BLOCK-iT RNAi Designer (www.invitrogen.com/rnai (accessed on 1 January 2021) Thermo Fisher Scientific, Waltham, MA, USA) was used to predict suitable siRNAs targeting the cDNA sequences of EmTUBB3 (EmuJ_000202500). These siRNA sequences were manually rechecked relative to their position, target site, length of siRNA, nucleotide content and specificity of siRNA (off targets). The two selected siRNAs (siRNA-1 and siRNA-2) were synthesized by GenePharma Co., Ltd. (Suzhou, China). The sequences of the two siRNAs targeting EmTUBB3 were as follows: siRNA-1, 5′-CAGAGAGGAGUAUCCAGAUCGUAUU-3′ fluorescein; siRNA-2, 5′-CCUACCAAAGCUGUACGAUUCUUGA-3′ fluorescein. To determine the transfection efficiency, another negative (nonsilencing) control siRNA with the FAM fluorescence label was purchased from GenePharma Co., Ltd. (Suzhou, China) as an unrelated siRNA.

### 2.3. siRNA Delivery to E. multilocularis PSCs

We established four siRNA groups: two TUBB3 siRNA-treated groups (siRNA-1/2), a negative control siRNA group (NC), and a control group without RNAi intervention (untreated). Electroporation was used to deliver siRNA into PSCs in vitro. In brief, 2000 PSCs were washed three times with RNAi electroporation buffer (150 mM sucrose, 27 mM Na_2_HPO_4_, pH 7.5) and then resuspended in 100 μL electroporation buffer containing FAM-labelled control siRNA to a final concentration of 5 μM in a 4-mm electroporation cuvette. Electroporation was performed at 100 V, 800 µF and with an exponential decay pulse (Gene Pulser II, Bio-Rad, Hercules, CA, USA). After incubation at 37 °C for 10 min, 1 mL culture medium was added, and the PSCs were transferred to 24-well plates for an additional 2 h of incubation at 37 °C in 5% CO_2_ in the dark. Then, the parasites were washed three times with PBS and observed under a confocal microscope (TCS SP8, Leica). The experiment lasted 15 days, and half of the medium was changed every 3 days [25]. Each treatment was carried out in triplicate, and experiments were repeated twice using PSC samples collected at different time points.

### 2.4. Viability of PSCs Treated with siRNA Targeting EmTUBB3

The treated PSCs were incubated for 15 days in a RPMI 1640 medium (Invitrogen, Carlsbad, CA, USA) supplemented with 10% (*v*/*v*) heat-inactivated FBS (Invitrogen, Carlsbad, CA, USA), 100 U/mL penicillin and 100 μg/mL streptomycin (Thermo, Waltham, MA, USA) at 37 °C in an atmosphere of 5% CO_2_. The viability of the treated PSCs was evaluated every day by counting the number of PSCs that were stained with 0.1% (*v*/*v*) methylene blue [27]; the dye was applied only once per sample. Additionally, the number of evaginated PSCs per 100 PSCs and the number of PSC contractions per minute were assessed [31] under a light microscope (CKX41, Olympus, Japan).

### 2.5. Quantitative Real-Time PCR (qRT–PCR)

PSC samples were collected to determine the effects of RNAi on the expression of EmTUBB3 on days 3, 6, 10 and 15. Total RNA was extracted using TRIzol reagent (Invitrogen, Carlsbad, CA, USA) according to the manufacturer’s instructions. After treatment with DNase I (Thermo, Waltham, MA, USA) for 30 min at 37 °C to remove genomic DNA contamination, the RNA samples were transcribed into cDNA using a reverse transcription kit (TaKaRa Bio, Dalian, China). qRT–PCR was performed using 2 μL of 1:5 diluted cDNA and SYBR Green Real-time PCR Master Mix (TaKaRa Bio, Dalian, China) and run in a thermocycler (ABI Prism 7500, Bio-Rad, Hercules, CA, USA). The following specific primers for EmTUBB3 and EF-1α (internal control) were synthesized by Sangon Biotech (Shanghai, China): EmTUBB3, 5′-AGGCTTGCGACTGCTTG-3′ (forward), 5′-CCGTGTCCGACACCTTAGGT-3′ (reverse); EF-1α, 5′-TTTGAGAAAGAGGCGGCTGAGATG-3′ (forward), 5′-TAATAAAGTCACGATGACCGGGCG-3′ (reverse). The qRT–PCR protocol was comprised of 39 cycles at 95 °C for 5 s, 60 °C for 10 s and 72 °C for 30 s. Each reaction was run in triplicate, after which the average threshold cycle (Ct) was calculated per sample and the relative expression of the genes was calculated using the 2^−ΔΔCT^ method [32].

### 2.6. Morphological Observation

The effects of RNAi on the morphological and biological changes in PSCs were evaluated by scanning electron microscopy (SEM) and transmission electron microscopy (TEM). For SEM, the PSCs were fixed with 3% glutaraldehyde in 0.1 M sodium cacodylate buffer (pH 7.2) at 4 °C for 24 h. After rinsing for 20 min 4 times with PBS, the samples were postfixed in 1% osmium tetroxide for 1 h, dehydrated in a graded series of ethanol and acetone and critical-point dried with liquid CO_2_ [33]. The samples were sputter-coated with gold-palladium and examined on JEOL JSM 6700F (accelerating voltage 1.5 kV) and LEO-1420 (accelerating voltage 15 kV) instruments.

For TEM, the PSCs were fixed with 2.5% glutaraldehyde in cacodylate buffer at 4 °C for 24 h, washed overnight in 0.1 M sodium cacodylate buffer at pH 7.4, postfixed in 4 °C 1% OsO_4_ in the same buffer for 1 h, dehydrated in a graded series of acetone and embedded in Spurr’s epoxy resin within BEEM capsules [33]. Ultrathin sections (60–90 nm in thickness) were cut on a Leica Ultracut UCT ultramicrotome, placed on copper grids, and stained with uranyl acetate and lead citrate according to Reynolds [34]. The grids were examined with a JEOL JEM 1010 TEM instrument operating at 80 kV.

### 2.7. Statistical Analysis

All data are presented as the mean ± standard deviation (SD). Statistical comparisons between and within groups were performed by analysis of variance (ANOVA) using GraphPad Prism 8.3.1 software (GraphPad Software Inc., San Diego, CA, USA). Statistical significance was considered at *p*-value < 0.05.

## 3. Results

### 3.1. TUBB3 Expression in E. multilocularis PSCs after Treatment with siRNA

**Figure 1:**
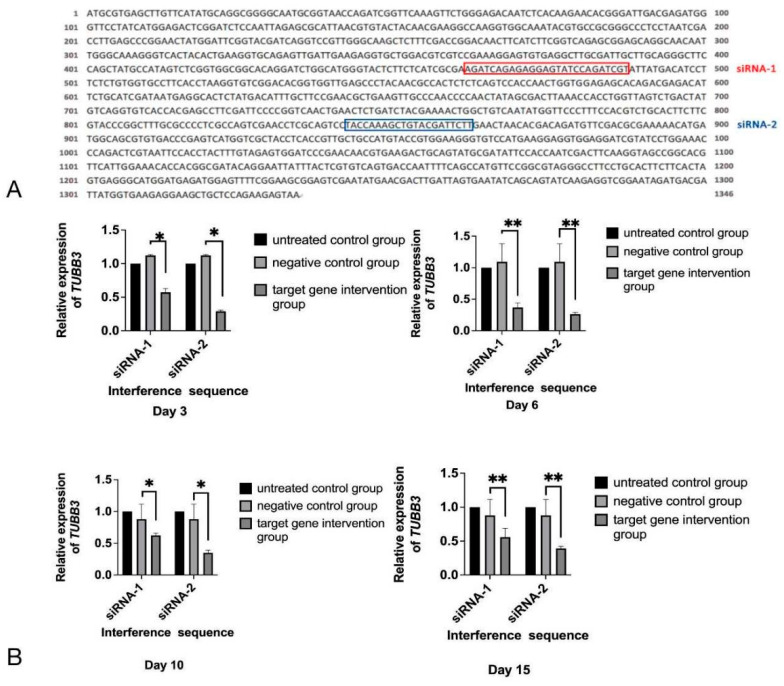
siRNA targeting EmTUBB3. (**A**) Sequence of EmTUBB3 and target sites of siRNA-1 and siRNA-2. (**B**) Relative expression of *TUBB3* in *E. multilocularis* PSCs treated with siRNA-1 and siRNA-2 at different time points. * *p*-value < 0.05, ** *p*-value < 0.01.

siRNA-1 and siRNA-2 were predicted to target sequences of EmTUBB3 mRNA (Figure 1A), which was confirmed by the evaluation of the relative expression of EmTUBB3, as shown in Figure 1B. After treatment with siRNAs, the expression of EmTUBB3 was significantly reduced. On day 3, compared with that in the electroporation control group, the EmTUBB3 suppression rates for siRNA-1 and siRNA-2 were 43% and 71%, respectively (*p*-value < 0.05). In addition, on days 6, 10 and 15, the EmTUBB3 suppression rates for siRNA-1 and siRNA-2 by 63% and 74% (*p*-value < 0.01), 38% and 65% (*p*-value < 0.05), and 44% and 61%, respectively (*p*-value < 0.01) (Figure 1B).

### 3.2. Viability of and Morphological Changes in PSCs Treated with siRNA-1 and siRNA-2

**Figure 2:**
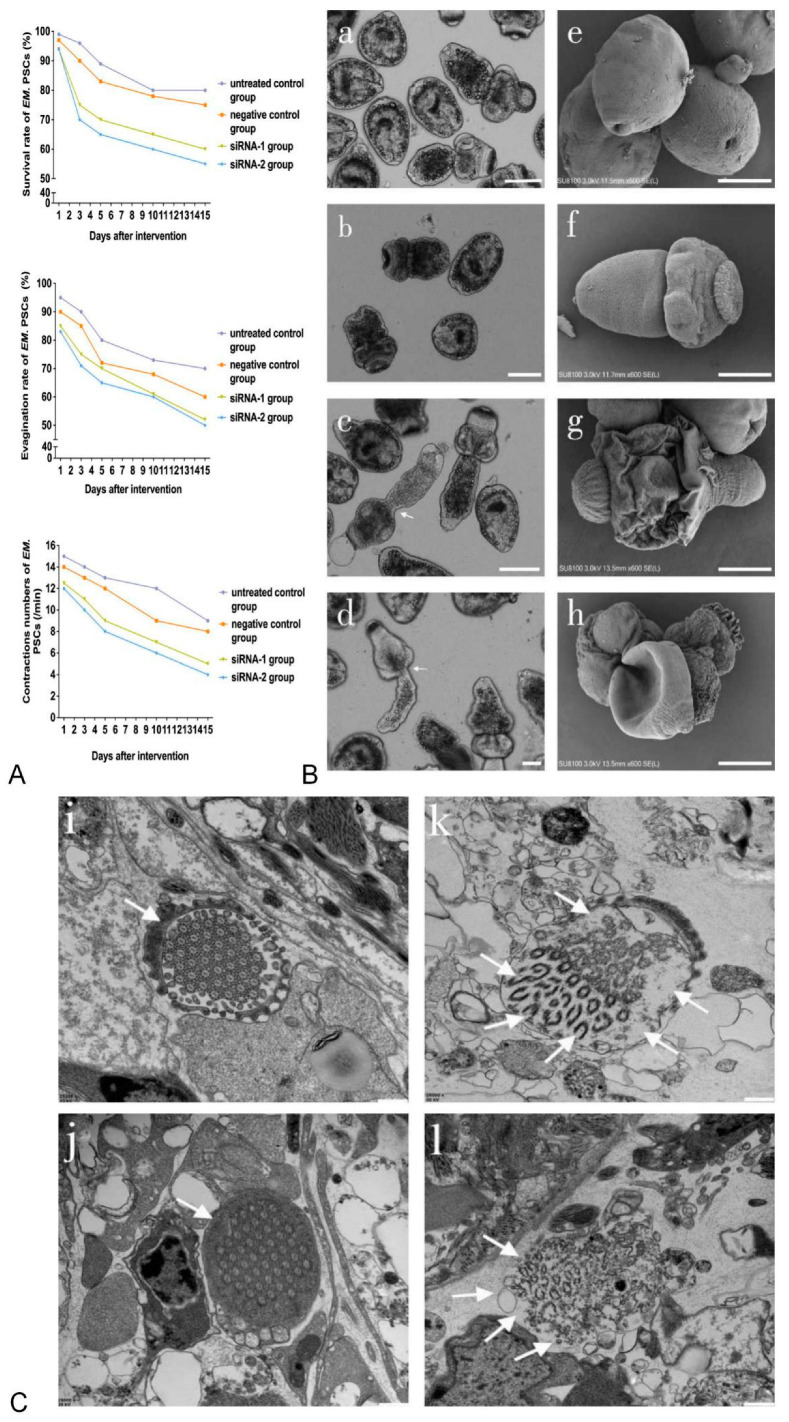
Biological changes in *E. multilocularis* PSCs treated with siRNA-1 and siRNA-2. (**A**) Biological changes in *E. multilocularis* PSCs treated with siRNA-1 and siRNA-2 at different time points. (**B**) Biological changes in *E. multilocularis* PSCs on day 15. Images a–d show the appearance of PSCs under a light microscope. Images e–h show the tegument surface under SEM. Images a and e are the untreated control group; b and f are the negative control group; c and g are PSCs treated with siRNA-1; d and h are PSCs treated with siRNA-2. Scale bar = 100 µm (**a**–**d**), 50 µm (**e**–**h**). The arrows represent irregular tegument shrinkage. (**C**) Ultrastructural changes in flame cells of *E. multilocularis* PSCs on day 15. Untreated control group (**i**) and negative control group (**j**), the arrows represent intact flame cells. siRNA-1 treated group (**k**), the arrows represent flame cells with cracked cilia enclosed in the cell body, empty internal cavities in the cytoplasm and ruptures of the microtubule structure. siRNA-2 treated group (**l**), the arrows represent flame cells lacking a cell membrane structure. Scale bar = 500 nm.

As shown in Figure 2A, the viability, evagination rates and numbers of contractions in siRNA-treated PSCs were time-dependent. On day 15, compared with those in the negative control group, the viability and evagination rates of siRNA-1- and siRNA-2-treated PSCs decreased to 60% and 55% (*p*-value < 0.01) and 52% and 50% (*p*-value < 0.01), respectively. In addition, the numbers of body contractions in siRNA-1- and siRNA-2-treated PSCs were reduced to five and four contractions per minute, compared with nine contractions in the controls, respectively (*p*-value < 0.01). The changes in the siRNA-treated PSCs, including morphological abnormalities such as irregular tegument shrinkage, roughness and shedding of axe-shaped hooks, were found to occur mainly in the tegument surface (Figure 2B, c d arrow and g h). However, these phenomena were not observed in the untreated controls, which had a smooth outer surface (Figure 2B(a,e)). Ultrastructural changes in the flame cells of the *E. multilocularis* PSCs treated with siRNA-1 and siRNA-2 are shown in Figure 2C. In particular, in both siRNA-1- and siRNA-2-treated PSCs, the structures of the cilia enclosed in the cell body were cracked, with empty internal cavities in the cytoplasm of the flame cell and ruptures of the microtubule structures (Figure 2C, k l arrows). In addition, siRNA-2-treated PSCs were lacking a cell membrane in the flame cell structure (Figure 2C, l arrows).

## 4. Discussion

RNAi has been successfully used to silence specific genes to elucidate their function and assess their therapeutic value in the management of conditions caused by infection by helminths, such as turbellarian [21], trematodes [35] and monogeneans [36]. However, there is limited information about its application in cestodes [25,27,31,37,38,39]. In a previous study, knockdown of the *elp* and *14-3-3* genes in *E. multilocularis* PSCs was successfully performed by the RNAi method [25]. In the present study, to promote the development of novel anthelmintic drugs based on tubulin gene targets, we successfully used electroporation to transfect *E. multilocularis* PSCs with TUBB3-specific siRNAs.

In the present study, we interfered with EmTUBB3 expression and observed the viability and morphological and biological changes in *E. multilocularis* PSCs resulting from RNAi. We found that the tegument surface of siRNA-treated PSCs exhibited irregular shrinkage, roughness and shedding of axe-shaped hooks. The morphological changes were similar to those in studies carried out by Mousavi et al. [31], who found that silencing expression of the *E. granulosus* tetraspanin gene resulted in reduced viability, body contractions and PSC evagination, leading to irregularities and malformations in tegument configuration. Tegument disruption may occur due to surface-renewal failure. Microtubule-based vesicle traffic from cytons to the syncytial layer provides a continuous renewal of the tegument surface. Since the tegument is a special type of epithelium, it seems that microtubule minus-ends orient to the outer surface [40]. The tegument is a syncytial layer with a plasma membrane that covers the whole worm and acts as an interface for host-parasite interactions. In platyhelminths, the tegument plays an essential role in different species in developmental processes involving the uptake of glucose, digestion, the secretion of substances, osmoregulation, water disposal, protection against enzymes and host immune responses [41,42]. Hence, destroying the tegument of *E. multilocularis* PSCs resulting from silencing EmTUBB3 is a potential method for disrupting the viability of this parasite.

Ultrastructural changes in flame cells, including the rupture of the cell membrane, a cracked cilia structure enclosed in the cell body, empty internal cavities in the cytoplasm of the flame cell and the rupture of the microtubule structure, were also found in the *E. multilocularis* PSCs in the siRNA-1- and siRNA-2-treated groups. This suggests that TUBB3 might play an important role in the formation of flame cells in *E. multilocularis* PSCs. In cestodes, flame cells are ciliated cells and the basic units of the protonephridial system (PS) of invertebrates. In parasitic platyhelminths, PSs play an important excretory role that allows the parasite to conserve water, eliminate salts and survive in the intestine or body cavity of the host; accordingly, they act as osmoconformers [43]. Parasites need to maintain, within physiological limits, the balance of osmotic pressure in their tissues to that of the host environment [43]. Under TEM, the flame cells of several helminths exhibit similar morphology, with a single nucleus and classical cilia with 9 + 2 axonemes [44,45], consisting of microtubules with alpha-tubulin and beta-tubulin dimers. Cestodes do not have a digestive system, so the presence of tubulin is important for their adaptation to and survival inside of hosts because the absorption-excretion-secretion system permits the continuous absorption of nutrients, as well as effective waste elimination [43,46]. For ciliated flame cells, the microtubule is functionally necessary [47], as described previously. Research on the cytoskeletal proteins of flame cells has demonstrated the presence of tubulin in flame cell cilia in the cestode *Gymnorhynchus* [48] and the trematode *Schistosoma mansoni* [49]. Since microtubules are the main cytoskeletal proteins that maintain flame cell cilia function, any alteration to them will induce malfunctioning of the excretory system, as was shown after the in vitro treatment of *Taenia crassiceps* [50]. The changes in flame cells in *E. multilocularis* PSCs verified that alterations to tubulins can influence the function of flame cells.

In the present study, the low expression of TUBB3 in *E. multilocularis* PSCs resulting in morphological and flame cell alterations is described for the first time. In the absence of applicable specific βIII-tubulin protein antibodies, we failed to complete the specific βIII-tubulin protein expression assay, which could elucidate the specific mechanisms involved in the process of tegument and flame cell changes; thus, further study is needed.

## 5. Conclusions

Silencing EmTUBB3 leads to the downregulation of expression of mRNA and is associated with reduced viability, a decreased evagination rate and a decreased number of contractions in *E. multilocularis* PSCs. In addition, βIII-tubulin is suggested to play a role in tegument formation and maintaining flame cell integrity in *E. multilocularis* PSCs. However, the detailed mechanisms are not yet fully understood. In the future, more evidence should be obtained to explore small-molecule inhibitors targeting EmTUBB3 for the treatment of AE. Hence, the present study serves as a basis for the development of novel applications in the management of echinococcosis in humans and animals.

## Data Availability

Not applicable.
